# Modelling aggregate exposure to pesticides from dietary and crop spray sources in UK residents

**DOI:** 10.1007/s11356-019-04440-7

**Published:** 2019-02-08

**Authors:** Marc C. Kennedy, David G. Garthwaite, Waldo J. de Boer, Johannes W. Kruisselbrink

**Affiliations:** 1 Fera Science Ltd, Sand Hutton, York, YO41 1LZ UK; 2 Wageningen University & Research, Biometris, Droevendaalsesteeg 1, 6708 PB Wageningen, The Netherlands

**Keywords:** Simulation model, Non-dietary exposure, Uncertainty, Cumulative assessment group, Pesticide usage survey

## Abstract

Human exposure to pesticide mixtures can occur from the diet and other sources. Realistic exposure and risk assessments should include multiple sources and compounds and include the relative hazards of the different compounds. The EU-funded Euromix project is developing new web-based tools to facilitate these calculations. A case study is presented that exemplifies their use for a population of UK residents, including exposure from crop-spraying. A UK pesticide usage survey provided information on real pesticide combinations applied to crops of wheat, potatoes, sugar beet and dessert apples. This information was combined with outputs from two alternative simulation models of spray drift to estimate dermal, oral and inhalation exposures of residents. These non-dietary exposures were combined with dietary exposure estimates using the Monte Carlo Risk Assessment software to produce a distribution of aggregated and cumulative exposures. Compounds are weighted by relative potency to generate a measure of overall risk. Uncertainty quantification was also included in the distribution of exposures. These tools are flexible to allow diverse sources of exposure and can provide important information to decision-makers and help to prioritise testing of pesticide mixtures. Including non-dietary sources changed the prioritisation of pesticide mixtures, when compared to dietary exposure alone.

**Electronic supplementary material:**

The online version of this article (10.1007/s11356-019-04440-7) contains supplementary material, which is available to authorized users.

## Introduction

The human population can be exposed to pesticides and other compounds from multiple sources and routes. In the production of raw agricultural commodities, combinations of pesticides are applied to crops using sprayers. Each individual in a population may be exposed to a mixture of pesticides from the residues present in their diet and through contact with sprayed pesticides. The residues in the diet will vary between individuals depending on the unique mix of items consumed, the origin of those items and the combination of pesticides applied to the crops. For an individual living near agricultural land, there is also a possibility of exposure from spraying. These aspects are highly variable and lead to complex distributions of mixtures within the population. For risk assessment, it is important to estimate the distribution of cumulative and aggregate exposures across the whole population as realistically as possible. Cumulative exposures are defined as those occurring from multiple compounds which contribute to a similar health effect. Such compounds are typically grouped into cumulative assessment groups (CAG) to aid the assessment of the effect. Aggregate exposures combine multiple sources including dietary and non-dietary sources. Both dietary and non-dietary sources of exposure can include mixtures, so aggregate exposure is considered as the combination from multiple cumulative exposure sources. The term *mixture* will be used to refer to a mixture of pesticides.

It is the responsibility of individual EU Member States to assess exposures within their respective countries, for regulation of pesticides with the aim of preventing harmful effects. It was recognised in the EU Regulation 1107/2009 that there is a need for improved general methods for integrating information on cumulative exposures, which can handle diverse exposure sources, models and data available within different countries.

The EU Horizon 2020 (H2020) project Euromix (no 633172, H2020-SFS-2014-2) is developing relevant methods and tools, building on earlier work in the EU Seventh Framework Programme (FP7) project Acropolis (van Klaveren et al. 2015, www.acropolis-eu.com). The aim of Euromix is to produce practical, general-purpose tools for risk assessment and testing strategies that can handle high-dimensional mixtures of the type found in practice. An important aspect of this is to prioritise relatively small numbers of compounds that occur together in reality, or those most likely to be harmful, so that resources can be targeted most effectively and animal testing can be minimised. Exposure assessment for mixtures is a first step and as far as practically possible should include multiple sources to provide the most accurate estimates of real exposures. The aggregate model described here generates detailed outputs on relative contributions to exposure from individual routes and compounds, which can be used as part of a risk assessment.

The tools developed in Euromix are available in the Monte Carlo Risk Assessment (MCRA) web-based platform (van der Voet et al. 2015, mcra.rivm.nl). The general aggregate model of MCRA was first described in Kennedy et al. (2015). MCRA has been further updated to incorporate the new features developed within the EuroMix project, particularly the ability to include many more compounds and to identify priority mixtures. For MCRA to be widely applicable, it is designed in a modular way, so that results from external software can be directly linked to the cumulative dietary exposure results.

Comparable models described in the literature include the Stochastic Human Exposure and Dose Simulation multimedia, multi-pathway models (SHEDS-MM) and the associated modules SHEDS-Residential (Glen et al. 2010) and SHEDS-Dietary. These were developed by the United States Environmental Protection Agency (EPA) (Zartarian et al. 2000, 2008, 2012) and combine databases and probabilistic modelling. Used together, SHEDS-Dietary, SHEDS-MM and SHEDS-Residential generate cumulative exposure distributions that account for residential exposure from spraying and from dietary sources, based on conditions and populations in the US. SHEDS-Residential has built-in scenarios for household spray activities. SHEDS-HT (Isaacs et al. 2014) is a high throughput extension of this model that allows for evaluation against the screening results of the EPA toxicity forecast (ToxCast) system. However, it is not clear how it could be adapted to include the specific exposures from agricultural crop spraying in the UK and does not incorporate EU dietary exposure data directly. MERLIN-Expo is a library of modules that can include multiple sources and processes developed within the EU FP7 project 4FUN (4funproject.eu). For integration, the chain of models is implemented using a standard proprietary software platform (Ecolego www.facilia.se) which allows for simple creation and visualisation of the model chain. It also includes functionality for standard sensitivity and uncertainty analysis. MERLIN-Expo only includes the models created by the developers and therefore does not allow general users to link their own models. The INTEGRA toolbox (Sarigiannis et al. 2014) is another software platform for aggregate exposure modelling (www.integra-lri.eu). It includes online databases of various parameters and integrates internal and external modelling of dose. The model provides a link to the ART (www.advancedreachtool.com) and TAGS (www.tags.cperi.certh.gr) systems and is especially suited to exposure from consumer products. NG CARES (caresng.org) is a cloud-based system that also includes dietary and residential exposure modelling. Users can input product application properties and chemical toxicity properties.

MCRA is designed for the dietary and compound monitoring databases in the EU, based on standard formats developed in collaboration with the European Food Safety Authority (EFSA) for dietary exposures. Consumption databases for individual country have been compiled and added to the system, so that a direct comparison between EU countries is possible.

A case study is presented below to illustrate how residential exposure to mixtures from crop-spraying in agricultural fields, aggregated with dietary exposures, can be addressed using MCRA. The UK adult population was considered. Two alternative non-dietary models were run in separate analyses. In the main analysis, the independent software developed in the EU FP7 Browse project (Butler Ellis et al. 2017, www.browseproject.eu) was used to generate non-dietary exposures for the relevant population. In a second analysis, the Bream2 model (Butler Ellis et al. 2018) was modified to quantify variability and uncertainty of residential exposures within the population. The purpose of demonstrating both analyses was to show how MCRA is flexible enough to include different types of non-dietary exposure models, including those that quantify uncertainty. The scenario used is consistent with that used in the analysis of Crépet et al. (2018), which compared cumulative dietary exposures between various EU countries. The new results show how the addition of non-dietary sources can change the selection of priority mixtures. It is also shown how usage data on the combinations of pesticides sprayed in UK crops can be incorporated to estimate cumulative non-dietary exposure. It is important to note that the results do not represent a real risk assessment, as the assumptions are based on limited information and would not necessarily be appropriate for that purpose. They are primarily designed to demonstrate the use of MCRA for aggregate exposure.

## Data and methods

### Risk scenario

**Fig. 1:**
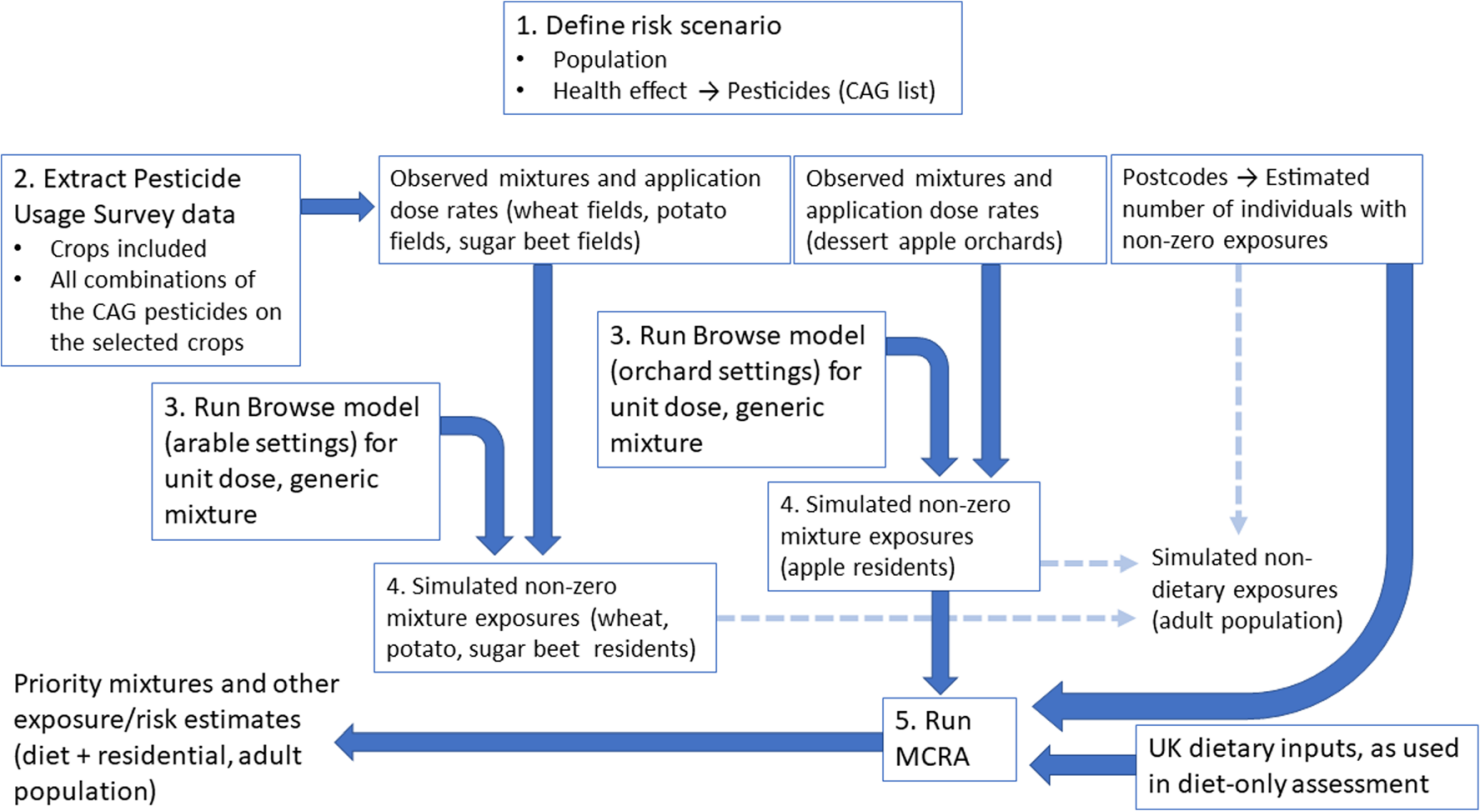
Process used to generate aggregate exposure estimates, combining Pesticide Usage Survey data and Browse model outputs within the MCRA simulation. Steps are numbered according to the order in which they are carried out, and arrows indicate the flow of information. Dashed arrows at step 4 show how intermediate non-dietary results are generated in addition to the overall aggregated estimates

Estimating the distribution of aggregate and cumulative exposures within a risk assessment requires a complex combination of data sources. Figure 1 provides an overview of the information flow and general processes carried out in sequence. Individual steps are explained in this and the following subsections. The first step was to define the risk scenario of interest, including the population, hazard and health effect. The population of UK adults aged 18–64 was considered for their exposure to pesticide mixtures. This decision was based on the availability of data. The chosen health effect was liver steatosis. Steatosis was previously used as a test case in Euromix to identify important mixtures in the dietary component (Crépet et al. 2018). Using consistent dietary assumptions and the same health effect allowed for a direct comparison between the diet-only and aggregate results. A list of pesticides that are relevant for this health effect was compiled from the literature. The starting list of 144 compounds considered relevant for steatosis was the same as used in this diet-only assessment and was compiled from three reports (Nielsen et al. 2012; RIVM et al. 2013, 2016).

### Pesticide usage data

The second step of the process depicted in Fig. 1 was to select some key crops to include in the analysis of non-dietary exposures, then to compile information about sprayed mixtures in these crops. The crops selected were wheat, sugar beet, potatoes and dessert apples. These make up approximately 47% of UK grown arable and orchard crops (Defra 2014). Wheat is the largest contributor to the total, and the other two arable crops were selected as examples with different patterns of pesticide use. Although they represent a small proportion of overall crops in the UK, dessert apples were also included as an example of an orchard crop. Information on orchards is treated separately in the estimation process (Fig. 1) as orchards are subject to different crop spray characteristics and modelling. Dessert apples were therefore included to demonstrate the ability of MCRA to incorporate exposure models of diverse spray model types. Inclusion of further crop types was not considered necessary for the exercise. The sub-population of UK adults with non-zero exposures was assumed to comprise those living adjacent to fields growing one of the selected crops.

UK pesticide usage was taken from the Pesticide Usage Survey (PUS) for 2014 (Garthwaite et al. 2015a,b). The PUS provides a stratified sample of UK farms detailing the amount of each pesticide^1^ applied in each field and spray round. Sampling was stratified by region and by farm size group, as different pesticide usage patterns are known to occur in different size groups. Each surveyed field has an associated raising factor that extrapolates from the field to the national level and adjusts the estimates to account for bias in the particular sampling strategy. The raising factors are derived separately for arable and orchard crops using standard statistical methods based on the number of farms surveyed relative to the total number of farms growing each crop type. Details are provided in Thomas (1999). Total areas of crops harvested in 2014 were also used in the calculation.

Usage data were extracted from the PUS for wheat, potatoes, sugar beet and dessert apples. All combinations of the steatosis CAG compounds sprayed per surveyed field were included. Naturally, the total amounts sprayed are closely linked to field size. Using information in the PUS, including sprayed area (ha), the dose rates (kg/ha) were derived separately for all component pesticides in the sprayed mixtures.

Another use of the PUS data was to estimate the number of individuals living near specific crop types. This was required to estimate the proportion of individuals assigned positive non-dietary exposure from crop spraying, and which of the crop-specific exposures are relevant. For example, it may be that fields with large populations nearby have distinct spray combinations. The number of individuals living adjacent to agricultural land in the UK has been estimated previously and used as part of a study by the Royal Commission on Environmental Pollution (RCEP 2005). The Centre for Ecology and Hydrology (CEH) estimated the number of residents living adjacent to arable and horticultural land in Great Britain (GB) based on 1998 data to be approximately 2% of the population (CEH 2005). The method used a stratified sample of square satellite images of different land types to estimate the length of field boundaries in the UK adjacent to residential housing and the average number of households per length of boundary in different land use types. Information from the 2001 Census of the Office for National Statistics (ONS 2001) was then used to estimate a simple average of occupants per household. Resident numbers were extrapolated to a GB level by scaling up the land sample areas in proportion to their full geographic areas. The calculation was repeated with 1000 bootstrap samples to produce a 95% confidence interval (1–1.5 million) on the total number of residents.

An alternative approach was used in the current study that linked the PUS sampled fields with the residents living nearby. Data from the more recent UK Census (ONS 2011) were used to link the postcodes of each field with the number of individuals living in the same postcode area. The data were accessed using the geoConvert website (UK Data Service 2016). This is a pragmatic but simplistic approach because individual postcodes link to postal areas (typically roads) rather than farmland. However, it was the only available information on residents in each area and allowed for the surveyed fields to be linked explicitly to a spatial area. This allows for spray patterns in particular crop types or fields to be associated with the type of residential area. This assumption, that all individuals in the same postcode live beside sprayed fields, leads to conservative estimates for the proportion of the population affected. Where no postcode information was provided, the number of individuals was randomly assigned from all fields of the same crop type. The same factors used to raise farm areas to UK totals were applied, per sampled field, to raise the number of individuals to produce UK totals. The UK population was assumed to be 63.2 million with 82.4% of these being adults (ONS 2001). In the MCRA input, the proportion affected is relative to the corresponding *subpopulation* in the survey, which is adults.

### Browse model for resident exposure

Step 3 in the process shown in Fig. 1 was to generate simulated resident exposures using the Browse model. Browse is a probabilistic model that predicts pesticide exposure resulting from crop spraying. Probability distributions are included to quantify variability in some of its input parameters (wind speed, wind angle and boom height) with the user input values taken as averages. Monte Carlo sampling is used to generate samples from the implied probability distributions of the output. Multiple realisations within the probabilistic simulation represent variations in the input conditions. Consequently, the resulting output realisations aim to capture variation in real-world exposures (mg/day) per unit of spray dose. In each case, dermal, oral and inhalation exposures were generated separately. The dermal and oral routes can include direct deposition of spray drift on the skin, subsequent hand to mouth contacts and indirect contact with ground deposits. Inhalation can occur from vapour released following spraying.

Browse requires various parameter settings and the level of refinement in a scenario can be varied according to the level of detail available. Defaults are available based on a guidance document produced by EFSA (2012a), which tend to be conservative as required for risk assessments. These defaults have been built into Browse. The selected crops include three arable and one orchard crop. These have different spray properties and are handled by distinct Browse model algorithms, so the process was carried out separately for an arable scenario and for an orchard scenario. The input settings defining the two scenarios are listed in Table 1. Other parameters not listed here were kept at the built-in default values. Implications for the overall level of conservatism in the output are discussed in section “Discussion” and in the supplementary material.

**Table 1:** Browse model settings used for the arable and orchard crop scenarios. Arable settings were used for wheat, potatoes or sugar beet and orchard settings were used for dessert apples

Browse parameter and units	Arable settings	Orchard settings
Long-term assessment period (months)	3	3
Concentration of active ingredient in product (g/l)	250	250
Product dose (l/ha)	4	4
Sprayed volume rate (l/ha)	200	500
Forward speed (km/h)	12	6
Spray quality	Medium	Very fine
Drift reduction	None	None
sprayer type	Vehicle mounted boom sprayer, no cabin	Vehicle mounted sprayer, axial fan sprayer
Number of passes of the sprayer	3	–
Boom width (m)	24	–
Boom height above crop (m)	0.7	–
Climate scenario for volatilisation	Central–Hungary	–
Growth stage	–	Dormant
Dermal, oral, inhalation factors (fraction)	1	1

In the current work, the focus is on long-term daily average exposures for adult residents, as steatosis is a chronic health effect. The period assumed for chronic exposure was 3 months, representing a typical spraying season.

As represented in the Browse model (Butler Ellis et al. 2017; Kennedy and Butler Ellis 2016), random simulated exposures are considered for a resident aged 18–64 who is living between two fields sprayed with the same product. The individual is always present directly downwind of the spray and is located at random distances uniformly distributed between 2 and 20 m from the edge of the sprayed field. Three passes of the sprayer are assumed, where each pass is further away from the resident than the previous one. The combined total spray for each spray round is assumed to be applied in a single application. This single application assumption is a simplification but only affects vapour calculations but is a simplification.

The selected climate scenario affects the vapour calculations only. Browse includes the option of three diverse scenarios corresponding to the northern, central and southern EU regulatory zones. Air temperature is known to be a key driver for volatilisation, so in each zone a conservative climate record was selected at a site with the 90th percentile mean air temperature over a growing season. The UK falls within the selected central zone. Further assumptions about residents and crop spraying conditions followed the default conservative selections of the Browse model. A more detailed explanation of the input parameters is available in Butler Ellis et al. (2017).

Browse was used to generate the maximum available number of simulations (17,500) of exposure for a unit dose rate (kg/ha) of a generic mixture. The combination of 250 g/l for concentration of active ingredient in the product and 4 l/ha product dose leads to the required unit dose of 1 kg/ha. Browse does not handle pesticide mixtures, so these were used as a base sample of standardised exposures, to be rescaled in a subsequent step.

### Simulating from the probability distribution of exposures per crop

Dermal, oral and inhalation exposures are calculated in the Browse bystander and resident model using dose (kg applied per hectare) as a simple multiplicative factor. This means that the single large sample from the distribution of exposures with dose = 1 can be reweighted to approximate the actual exposures for multiple fields and pesticides. Step 4 in the process (Fig. 1) was therefore to combine the unit-dose Browse simulations with real dose rates from the PUS, collected in step 3, to produce simulated exposures for those individuals with non-zero exposures. This was achieved by simply multiplying the corresponding dose amounts for a randomly assigned PUS field for individual *i* and compound *j*. The assigned field for individual *i* determines the relevant mix of compounds and their product doses. This is appropriate as the individual is assumed to live beside fields of only a single crop type. The field-specific exposures generated by this process reflect the actual mixtures applied, including the relative dose rates. The algorithm to calculate exposure distributions for chronic exposure is outlined below. This process was repeated for each of the four crop types:

A fixed number (*I* = 2000) of positive individual exposures (mg/day) were simulated. Each of these individuals was randomly assigned to a PUS surveyed field of the appropriate type. For individual/field *i*, the number of unique compounds sprayed during the year is denoted by *J*_*i*_. For each *i* = 1, … , *I* and compound *j* = 1, … , *J*_*i*_ a simulated Browse chronic exposure set

$$ {\boldsymbol{c}}_{ij}={\left({c}^{Dermal},{c}^{Oral},{c}^{Inhalation}\right)}_{ij} $$

is selected at random from the 17,500 generated for the unit product dose and generic mixture. The three components of ***c***_*ij*_ are correlated due to the shared process models used in Browse to estimate them. Each unit dose simulation was taken to represent a randomly selected resident living beside a field growing that crop. The independent selection of Browse exposures per compound allows for the possibility that each compound was sprayed from a separate spray event, which is necessary because sprayed amounts of a given compound are aggregated per field and are not necessarily sprayed together as a tank mix at every spray round. The averaging for chronic exposure has already been performed within Browse, as explained above, as a daily average dermal, oral and inhalation exposures over a 3-month period. The concept of a survey in MCRA inputs allows for multiple sets of non-dietary exposures to be linked to different sub-populations. Each crop was assigned a unique survey number, so that, for example, exposures for residents living near orchards are distinguished from those living near wheat. Each crop type effectively generates a separate survey of simulated exposures, with 2000 individuals and a varying number (*J*_*i*_) of compound records per individual *i*. For the considered types (wheat, potatoes, sugar beet, apples), the simulated surveys of non-dietary exposure were combined into a single non-dietary exposure MCRA input file in the correct format (mcra.rivm.nl/Support/DataFormatsManual). The simulated positive exposures are only relevant for the specific sub-population of individuals that live next to a field of a given crop type. For all crop types, the percentages of individuals, derived in step 2, were therefore used to set a zero-proportion parameter. These parameters were included in the MCRA non-dietary exposure input file in addition to the sub-population properties. Each person from the simulated population of residents was assigned a unique identifier and was linked to just one of the crop-specific exposure surveys. Linking each individual to a single crop type in the input files ensures that the same individual cannot be assigned exposures from multiple crops. The use of *I* = 2000 is arbitrary but large enough to capture random variation within the PUS records of each crop type.

Prior to the final integration in MCRA, some intermediate summaries were generated to investigate the mixtures and single pesticides to which residents may be exposed. Each simulated data point was weighted by the raising factor that represents the relative number of UK residents associated the corresponding field (section “Pesticide usage data”).

### Linking non-dietary exposures and dietary exposures in MCRA

The combined file was used as the NonDietaryExposure table input to MCRA as part of an aggregate exposure assessment. Aggregate and cumulative options were selected in MCRA for the model type. Other settings were chosen to be consistent with the diet-only assessment of Crépet et al. (2018) including the choice of the optimistic model for dietary pesticide residues. The alternative, pessimistic, model for pesticide residues in the diet was designed to be conservative by giving an upper bound on true dietary exposure. This has been found in some cases to generate implausibly high exposures that are not useful for aggregate exposure assessments, so it was not considered.

UK dietary consumptions were extracted from the National Diet and Nutrition Survey (NDNS) (Henderson et al. 2002) and recoded to use the FoodEx1 coding system (EFSA 2011). FoodEx1 was developed by EFSA as a standardised system for classifying food, which allows data from different member states to be harmonised. It allows for consistent analysis and comparisons between countries. Food items are organised according to a hierarchy in which broad general food types such as bread, fish or dairy can be further classified using more specific sub-types. A merged dataset of residue concentrations was used, as described in Crépet et al. (2018), which used the Standard Sample Description 1 (SSD1) format (EFSA 2010). Specific UK concentrations were not available for this study, but the merged dataset provided a large sample size (127 pesticides from the steatosis CAG and over 3 million values from 204 commodities). This use of a multi-country residue dataset is consistent with the consumption of internationally traded commodities in the UK.

During the MCRA internal simulation, dietary survey individuals are either assigned a zero non-dietary exposure, with probability equal to the zero-proportion parameter, or were linked randomly to one of the non-dietary exposure records. Cumulative effects of the multi-compound exposures are integrated to produce a single measure using a relative potency factor (RPF) to weigh each compound relative to a chosen reference compound. Flusilazole was used as the reference compound, based on a prioritisation exercise that first identified the compounds with long-term studies and with the most reliable NOAEL or LOAEL, for fatty changes in liver, then from the remaining compounds selecting the one with most studies showing liver effects. More details are given in Crépet et al. (2018). The RPF weighting of individual mixture components accounts for the relative hazards and allows for an analysis of the overall risk. RPFs were estimated as the ratio of the NOAEL of the reference compound divided by the NOAEL of the individual compound or by the alternative approximation LOAEL/3, if no NOAEL was available. The NOAEL, LOAEL limits were taken from Nielsen et al. (2012) and RIVM et al. (2013, 2016). This use of RPFs is based on an assumption of dose additivity, which is usually conservative in the sense of over-estimating the mixture effect, and has been recommended as the default model for regulatory assessment of cumulative risks of pesticides in the EU unless evidence exists of non-additive (synergistic or antagonistic) effects (ESFA 2013). It should be noted that RPF weighting takes place within MCRA after the linking of dietary and non-dietary samples and aggregation, so the non-dietary exposure input files provided to MCRA are always unscaled for potency. Similarly, non-dietary exposures are unscaled (mg per individual), so the appropriate bodyweight scaling was applied within MCRA after calculation of the aggregate exposure.

### Quantifying uncertainty in non-dietary exposures

The Browse model simulates the effect of natural variability in its input parameters, such as wind direction or wind speed, but does not characterise uncertainty (lack of information). Some non-dietary exposure models also include a measure of uncertainty in individual exposures. When running for multiple variable inputs, these can also be used to generate uncertainty in the full distribution of population exposures with a two-dimensional Monte Carlo (2DMC) approach. MCRA is designed to allow uncertainty and variability to be propagated into the aggregate exposure results. To illustrate this, a modified version of the Bream2 model was used to generate 1000 individual exposures repeated with 100 uncertainty realisations. Uncertainty is associated with unknown parameters of a sub-model that calculates the collection efficiency of a human bystander in contact with airborne spray. Further uncertainty is due to an unknown scale correction factor that is inferred from a combination of wind tunnel and field data (Butler Ellis et al. 2018). These parameters were estimated using a limited set of measurements, and the impact of the uncertainty should be assessed. The modification was necessary as the published Bream2 model uses fixed values for the uncertain parameters and therefore, like Browse, does not separately quantify uncertainty and variability.

For this illustration, only wheat was considered, as it is the most common UK crop. The PUS data for wheat were used in the same way as described above, including the partitioning of total spray amounts into the survey-based compound proportions for every spray event. Each simulated individual was assigned a single field and exposed via all spray events for that field. A 90-day period was assumed to scale the total exposures to average day exposures. Bream2 simulates ml of spray liquid from a reference flat fan FF110/03 nozzle with a fixed dose rate of 0.2 kg/ha (based on 200 l/ha spray volume and pesticide concentration of 1 g/l sprayed liquid). For comparison, the Browse model was set up to simulate a unit dose rate of 1 kg/ha. Therefore, the Bream2 standardised simulation results were multiplied by 5 then multiplied by the recorded dose from the individual PUS spray records to represent the distribution of exposures (ml/day based on the actual dose rate (kg/ha)). An input file was created for MCRA, including 100 uncertainty values for each simulated individual, and an uncertainty run was generated. The MCRA settings were the same as described in section “Linking non-dietary exposures and dietary exposures in MCRA” except that 100 uncertainty cycles were included. Dietary records were also resampled as part of the analysis, so that uncertainty was propagated into the dietary and non-dietary exposures. Within the non-dietary uncertainty file, each uncertainty realisation has a unique identifier. For each uncertainty cycle of the dietary assessment, the dietary exposures are paired with non-dietary exposures for a single randomly selected non-dietary identifier.

## Results

Two types of result are reported below. First, the simulated population of non-dietary exposures is presented. Secondly, the aggregate outputs from MCRA are summarised and compared with the corresponding diet-only exposures.

### Non-dietary exposure

Table 2 summarises the area of land covered and the percent of the UK adult population for each of the considered crops. These were estimated directly from the PUS data using the method described in section “Pesticide usage data”. The dominant crop type by grown area is wheat. Between 97 and 100% of grown crop area is treated with one or more of the compounds included in the steatosis CAG. Wheat fields included 29 of the compounds in the CAG for liver steatosis.

**Table 2:** Percentages covered by the compounds in the steatosis CAG list, for four crop types, based on UK Pesticide Usage Survey in 2014. Estimated percentage of UK population living nearby is also shown

Crop	Total grown area (million ha)	Percent of total UK arable and orchard area	Percentage of grown area including one or more compounds in the steatosis CAG	Adult residents near fields (percent of UK adult population)
Wheat	1.94	41%	99.54%	8.5%
Sugar beet	0.12	3%	100%	0.67%
Potatoes	0.12	2.5%	99.43%	0.8%
Dessert apples	0.005	0.5%	97.39%	0.09%

The total number of individuals represented in Table 2 is just over 10% of the UK adult population of 52.1 million. This is substantially higher than the interval produced by CEH where the 95% confidence interval was estimated as 967,280–1,479,150 residents (CEH 2005). This leads to a more conservative estimate of the true proportion of the population exposed to these pesticides due to crop spraying. The different estimates suggest that this may be around 5 times the true proportion.

**Fig. 2:**
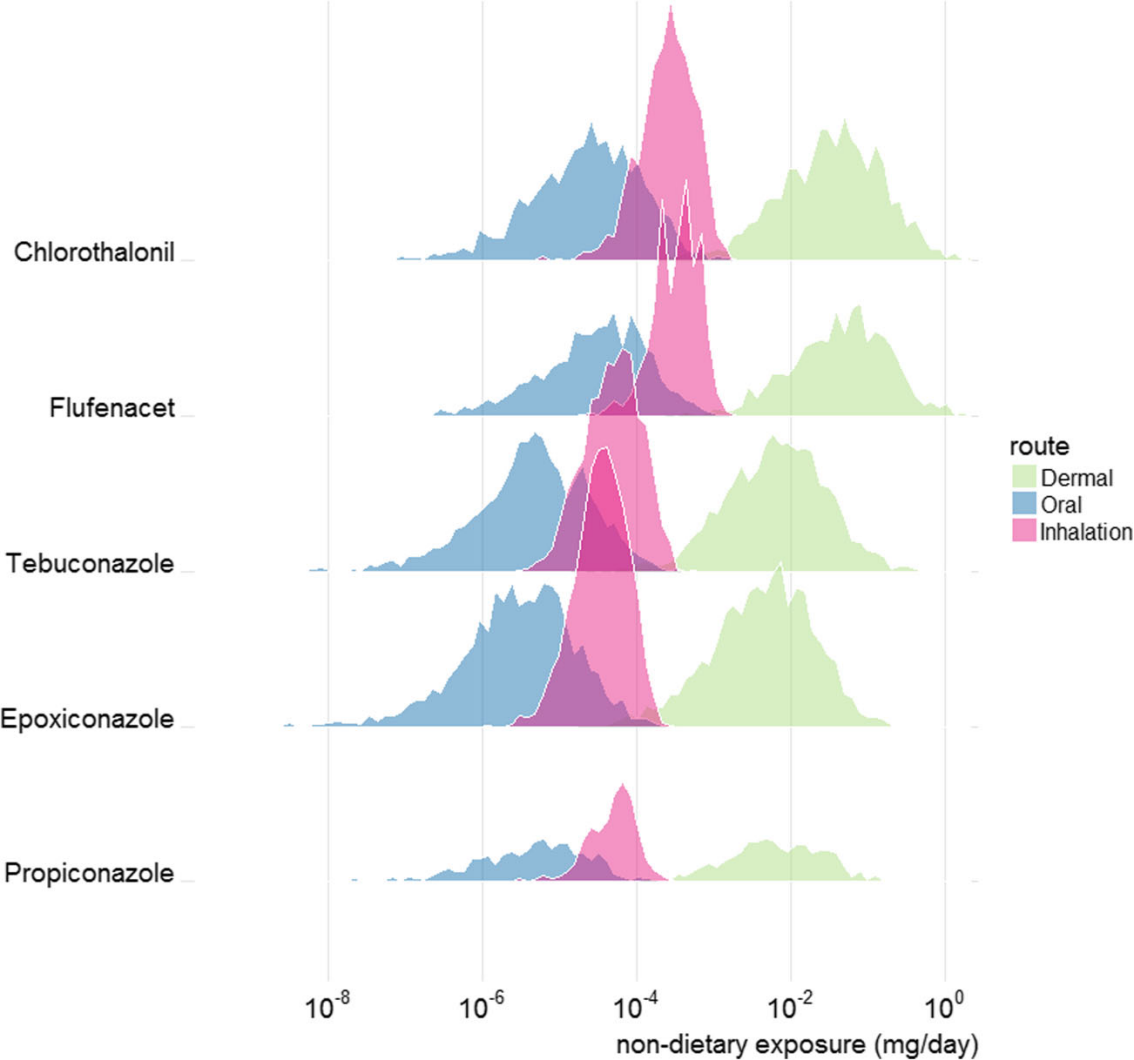
RPF-weighted non-dietary exposures generated using the Browse model for the five compounds with largest (population aggregated) dermal exposure, from the liver steatosis CAG list. These exposures represent non-zero simulated values associated with UK wheat fields in 2014. Units are mg/day flusilazole equivalents

**Fig. 3:**
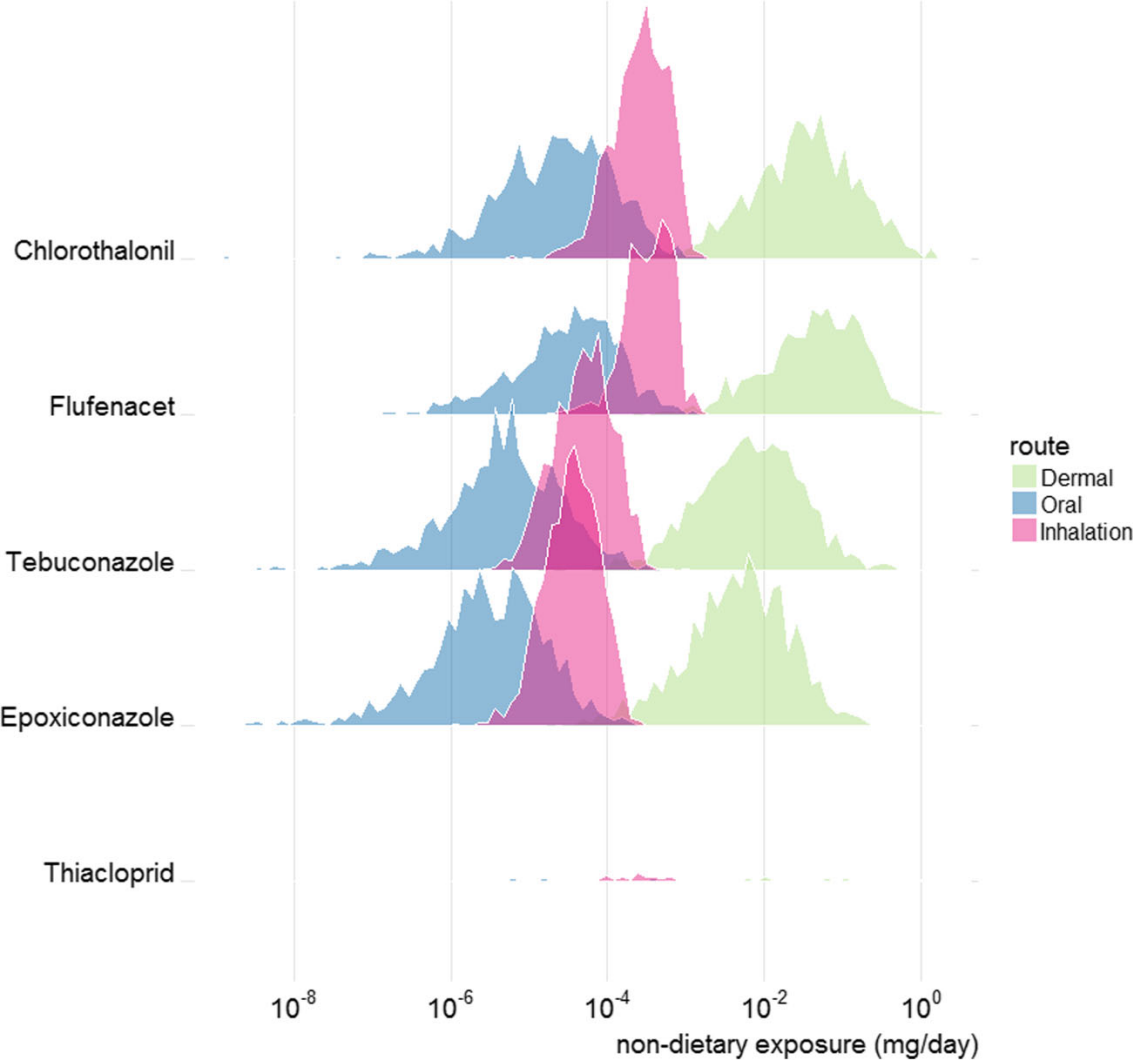
RPF-weighted non-dietary exposures generated using the Browse model for the five compounds with largest (population aggregated) dermal exposure, from the liver steatosis CAG list. These exposures represent non-zero simulated values associated with fields of wheat, potatoes, sugar beet or dessert apples in 2014. Units are mg/day flusilazole equivalents

Combining the simulated non-dietary surveys for the 4 crops produced an input file with 35,460 non-zero exposure records. These included 2000 fields for each crop type and within each of the 8000 fields/residents a mix of compounds according to the random selection from the PUS data. The average number of compounds per field was 4.4 but it ranged between 1 and 12. The combined number of compounds was 52. Example non-zero exposure results are displayed in Figs. 2 and 3. These show dermal, oral and inhalation exposures separately for the individual compounds contributing most to total exposure. Figure 2 relates to the most common crop (wheat) whereas Fig. 3 includes individuals exposed from spray activity in any of the four crop types. These provide information about the frequency and amount of exposure occurring in the simulations, for individual compounds in the CAG. For example, in Fig. 3, thiacloprid exposures are seen to occur less frequently than others, but thiacloprid can contribute significant amounts to the dermal exposure in some individuals. Thiacloprid appeared in spray records for potatoes and dessert apples but not for wheat. For all compounds, dermal exposure contributes the most to the total with inhalation contributing less and oral contributing the least. This ordering is produced by the Browse algorithm.

**Fig. 4:**
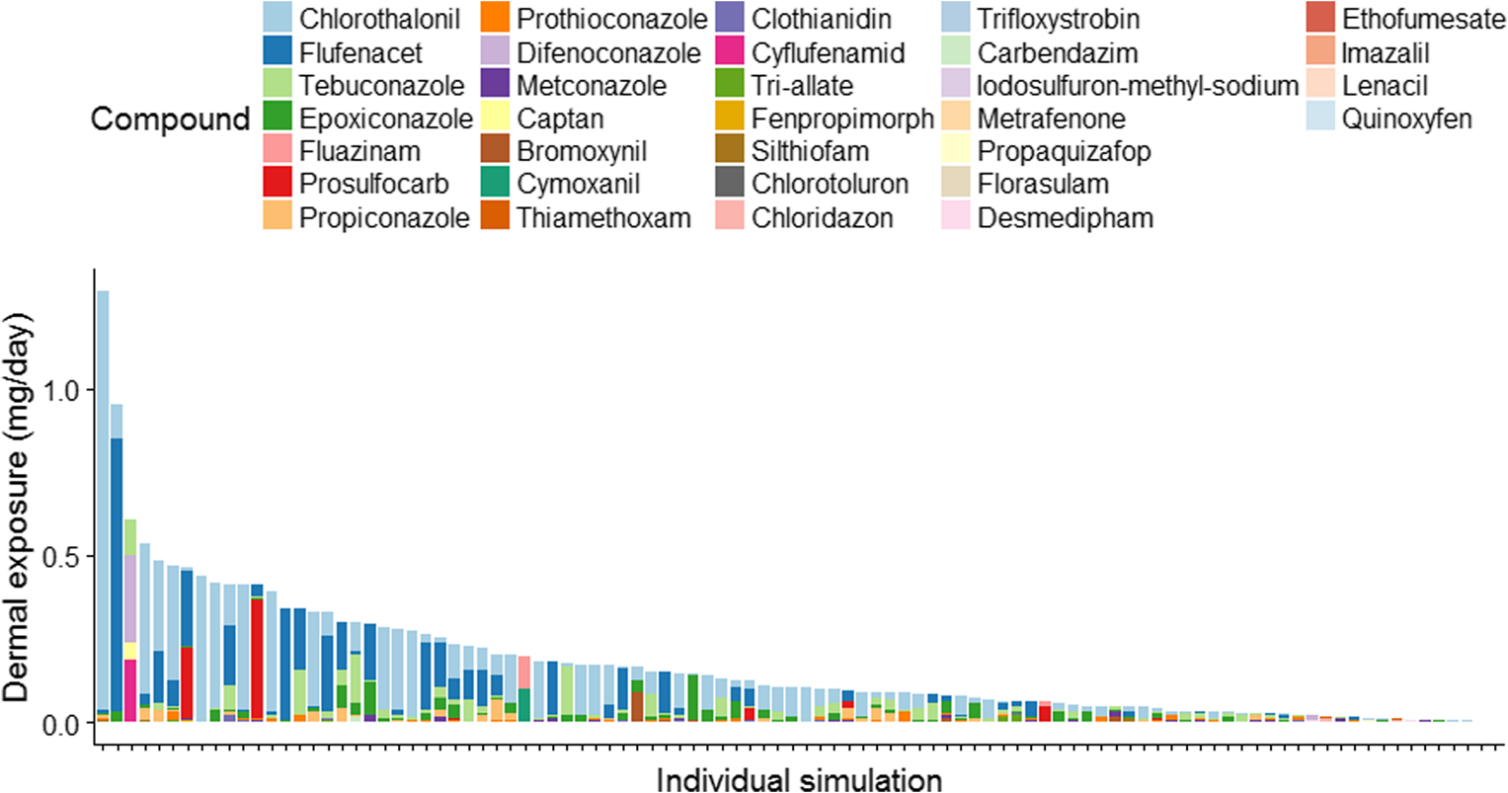
Mix of compounds for 100 simulated individuals, sampled from the positive exposure simulations in proportion to the population resident probabilities per crop type. RPF weighting is applied for each compound

Figure 4 shows the mixtures per individual, for 100 individuals randomly selected from the 8000 simulated, weighted using the probability of residence near one of the 4 crop types (Tables 2 and 3). These illustrate the range of typical mixtures to which the UK population may be exposed. Exposure related to dessert apples and potatoes do appear in the top 100 although the majority are from wheat, with 83 out of the top 86 exposures. Sugar beet appears in this list but at relatively low levels, since the RPF-weighted exposures are lower. For individuals living near wheat fields, the main compounds are chlorothalonil and flufenacet although mixtures involving prosulfocarb, tebuconazole, epoxiconazole and others also occur. For other crops, different compounds appear such as cyflufenamid, fluazinam, difenoconazole, propiconazole and captan. In the MCRA results presented below, these simulated results are carried forward into the aggregate assessment and included when identifying important overall mixtures.

**Table 3:** Population summaries of total dermal, oral and inhalation exposures generated for simulated individuals (chronic exposures in mg/day flusilazole equivalents) for UK residents near fields of wheat, potatoes or sugar beet, or dessert apple orchards. In each simulated individual, these are RPF weighted and are summed when multiple compounds are present

Route	Full population summary			Exposed sub-population summary		
	Median	Mean	P95	Median	Mean	P95
Dermal	0	0.01078	0.04377	0.00331	0.02765	0.12547
Oral	0	8.22e-6	3.48e-5	2.34e-6	2.11e-5	9.76e-5
Inhalation	0	4.02e-5	2.53e-4	2.66e-5	1.0e-4	5.04e-4
Total	0	0.01082	0.04387	0.00337	0.02778	0.12567

### Dietary and aggregate exposure

#### Exposure distributions

Estimated population percentiles of cumulative exposure from the diet-only and the aggregated sources derived from MCRA are shown in Table 4. The mean exposures are 0.2582 and 0.4629 (μg/kg-bodyweight/day), respectively. The largest differences between the diet and aggregate exposure distributions occur in the upper tail percentiles. This is due to the small proportion of individuals simulated to have a relatively high exposure from the non-dietary source. Table 5 shows the MCRA output representing the contribution of individual routes to overall aggregate exposure. There are 1724 individuals in the dietary consumption survey. Each of these has simulated non-zero exposure, but only 186 of them have a non-zero non-dietary exposure included. This is approximately as expected from the estimated proportion living beside a sprayed field (Table 2). Table 5 also shows that the main contribution to the population exposure is generally from the diet (58.71%). Dermal exposure is also an important contribution (41.12%) due to the higher levels simulated for the most highly exposed individuals.

**Table 4:** MCRA percentile calculations for dietary and aggregate chronic exposures within the UK adult population, based on the reference compound flusilazole

Percentage	Dietary exposure (μg/kg bw/day)	Aggregate exposure (μg/kg bw/day)
50	0.1924	0.2217
90	0.5345	0.7298
95	0.6674	1.443
99	0.9818	3.641
99.9	1.422	13.96
99.99	1.667	25.23

**Table 5:** MCRA output summaries for the different routes of exposure and their contribution to the aggregate exposure. Contribution is the expected relative contribution to the total exposure, averaged over multiple simulations

Route	Contribution (%)	individuals with positive exposure	Mean exposure all individuals (μg/kg bw/day)	Median all individuals (μg/kg bw/day)	p95 all individuals (μg/kg bw/day)	Mean exposure individuals exposure > 0 (μg/kg bw/day)	p95 individuals exposure > 0 (μg/kg bw/day)
Dietary	58.71	1724 (100%)	0.2546	0.1924	0.6674	0.2546	0.6674
Dermal	41.12	186 (10.8%)	0.1887	0	0.9988	1.7486	5.6917
Inhalation	0.15	186 (10.8%)	0.0007	0	0.0045	0.0062	0.0158
Oral non-dietary	0.03	186 (10.8%)	0.0001	0	0.0007	0.0013	0.0051

#### Most important compounds contributing to non-dietary exposures

Considering the RPF-weighted exposure simulations and the proportion of the population affected, a list of compounds making the greatest contribution to the total non-dietary exposure was compiled. The top 17 entries by route and compound are shown in Table 6. These 17 contributions amount to 98.7% of the total and are all dermal exposures. The top 6 entries contribute over 90%. Some compounds, such as captan, can have high average exposure for those exposed but have relatively small contribution to the population exposure due to a small RPF and low numbers of individuals affected.

**Table 6:** MCRA output showing main contributions, by exposure route and compound, to non-dietary exposure. The exposure columns are not RPF weighted, but the contribution column accounts for the weighting

Exposure route	Compound name	Contribution (%)	Mean exposure all individuals (μg/kg-bw/day)	Individuals exposed (%)	Mean individual exposure > 0 (μg/kg-bw/day)	RPF
Dermal	Flufenacet (RD)	44.38	0.221	4.87	4.533	0.408
Dermal	Chlorothalonil	24.41	0.904	6.90	13.093	0.050
Dermal	Tebuconazole	8.85	0.189	7.02	2.693	0.090
Dermal	Fluazinam	5.48	0.074	0.93	7.989	0.133
Dermal	Thiacloprid	4.26	0.025	0.12	21.290	0.442
Dermal	Prosulfocarb	2.86	0.322	0.99	32.654	0.018
Dermal	Prothioconazole (RD)	1.63	0.147	7.71	1.906	0.021
Dermal	Difenoconazole	1.37	0.023	0.41	5.761	0.113
Dermal	Metconazole	1.11	0.017	2.78	0.611	0.123
Dermal	Captan	0.87	2.932	0.17	1684.829	0.001
Dermal	Cymoxanil	0.87	0.088	0.99	8.910	0.018
Dermal	Epoxiconazole	0.81	0.101	7.77	1.299	0.016
Dermal	Triadimefon and triadimenol (RD)	0.61	0.001	0.17	0.849	0.589
Dermal	Dimethomorph	0.55	0.027	0.52	5.137	0.035
Dermal	Clothianidin	0.27	0.029	2.32	1.261	0.020
Dermal	Fenpropimorph	0.18	0.037	1.33	2.809	0.008
Dermal	Silthiofam	0.16	0.004	0.93	0.411	0.083

#### Mixture selection

The most important mixture as calculated in MCRA, considering dietary exposure only, is shown in Table 7 (see also Crépet et al. 2018). The algorithm used to identify the mixture uses the sparse nonnegative matrix underapproximation (SNMU) of Gillis and Plemmons (2013). The SNMU weights given in the table represent the relative importance of a compound in the mixture. For comparison, the mixtures identified from the aggregate exposures are listed in Table 8. The non-dietary exposure summaries are generally higher than the corresponding whole population summaries. This is because the summaries are with respect to the positive exposures only, so ignores most of the population who are not exposed through crop spray.

**Table 7:** Main mixture identified from the UK dietary exposure example, as generated by MCRA and reported in Crépet et al. (2018). The dietary survey included 1724 adults and this mixture explained 71.6% of the variance in chronic exposure. Mean, median, P5 and P95 are summaries of exposure for the adult population and are not RPF scaled. For dietary exposures, these are identical to the corresponding summaries for exposed individuals, as most individuals are exposed

Compound	RPF	SNMU weight	Mean	Median	P5	P95
Imazalil	0.13	76%	0.77	0.29	0.003	2.98
Dithiocarbamates	0.53	20%	0.19	0.16	0.022	0.48
Carbendazim and benomyl	0.20	1%	0.03	0.02	0.003	0.07
Cypermethrin	0.28	1%	0.04	0.03	0.010	0.08

**Table 8:** MCRA mixture selection output calculated using the aggregate exposure with RPF weighting. The exposure summaries mean, median, P5 and P95 are calculated for the subset of individuals with positive non-dietary exposure and are not RPF scaled

Mixture	Variance explained	Compound	SNMU weight	Mean	Median	P5	P95
1	47.5%	Flufenacet (RD)	97.4%	4.533	1.943	0.246	20.069
		Chlorothalonil	1.2%	13.093	4.246	0.304	51.178
		Tebuconazole	1.0%	2.693	1.035	0.073	10.018
		Dithiocarbamates (RD)^a^	0.3%	NA	NA	NA	NA
		Imazalil	0.02%	0.329	0.175	0.05	0.194
2	35.2%	Thiacloprid	97.6%	21.290	0.08	0.08	52.98
		Difenoconazole	2.3%	5.761	0.698	0.056	16.599
		Imazalil	0.1%	0.329	0.175	0.05	0.194
3	9.0%	Chlorothalonil	99.3%	13.093	4.246	0.304	51.178
		Tebuconazole	0.5%	2.693	1.035	0.073	10.018
		Dithiocarbamates (RD) ^a^	0.2%	NA	NA	NA	NA

^a^Dithiocarbamates exposure in the main mixture occurs from dietary exposure only, so non-dietary summaries are not available

The main difference is the appearance of some relatively high exposure levels for flufenacet, chlorothalonil and tebuconazole for a fraction of simulated individual values. Even though there are relatively few affected individuals, the mixture selection process captured these new compounds. The contribution from the diet affecting most of the population was not highlighted as an important mixture, except for a small contribution of imazalil and dithiocarbamates in mixture 1 (Table 8).

The main mixtures shown in Table 8 are dominated by single compounds, so for identifying the most important mixtures for toxicology testing, a more targeted approach is required. The maximum cumulative ratio (MCR, Price and Han (2011)) is used in MCRA to quantify the relative importance of mixture exposures, compared to single compound exposures. It is defined as the ratio (cumulative exposure/maximum single compound exposure) and is computed for each simulated individual. A suggested variation of the mixture prioritisation algorithm is to restrict attention to those individuals with MCR value above a threshold, thereby focusing on the subpopulation affected by genuine mixtures. Table 9 shows the main mixtures identified when using the subset of 631 individuals whose MCR exceeded the cut off value of 2, rather than the full 1724 individuals included in the dietary survey. In this case, the third mixture identified is closer to the dietary exposure (Table 7) which suggests that the dietary contribution becomes more relevant for prioritising mixtures with more than two component compounds. However, the non-dietary exposure is still the main driver in determining the important mixtures.

**Table 9:** MCRA mixture selection output using the aggregate exposure and MCR threshold of 2

Mixture	Variance explained	Compound	SNMU weight	Mean	Median	P5	P95
1	34.4%	Chlorothalonil	32.7%	0.5408	0.0048	0.0009	2.5445
		Tebuconazole	32.7%	0.2279	0.0079	0.0020	0.5645
		Prosulfocarb	14.2%	0.4287	0.0006	0.0000	0.0031
		Flufenacet (RD)	12.4%	1.1681	0.7869	0.1917	2.6961
		Triadimefon and triadimenol (RD)	7.8%	0.0265	0.0042	0.0009	0.1306
		Metrafenone	0.2%	0.0131	0.0003	0.0000	0.0027
2	24.7%	Difenoconazole	48.5%	1.1681	0.7869	0.1917	2.6961
		Captan	47.7%	0.5408	0.0048	0.0009	2.5445
		Cyflufenamid	3.8%	0.1833	0.0000	0.0000	0.8511
3	16.6%	Imazalil	48.7%	0.5566	0.2817	0.0033	2.0428
		Dithiocarbamates (RD)	43.7%	0.1510	0.1046	0.0156	0.4107
		Cypermethrin (RD)	2.5%	0.0380	0.0312	0.0105	0.0894
		Carbendazim and benomyl (RD)	1.9%	0.0261	0.0179	0.0027	0.0736
		Triadimefon and triadimenol (RD)	1.2%	0.0265	0.0042	0.0009	0.1306
		Deltamethrin (RD)	0.9%	0.0155	0.0115	0.0015	0.0431
		Thiacloprid	0.8%	0.0221	0.0055	0.0006	0.1059
		Flufenoxuron	0.3%	0.0008	0.0005	0.0001	0.0025

#### Uncertainty in aggregate exposure

**Fig. 5:**
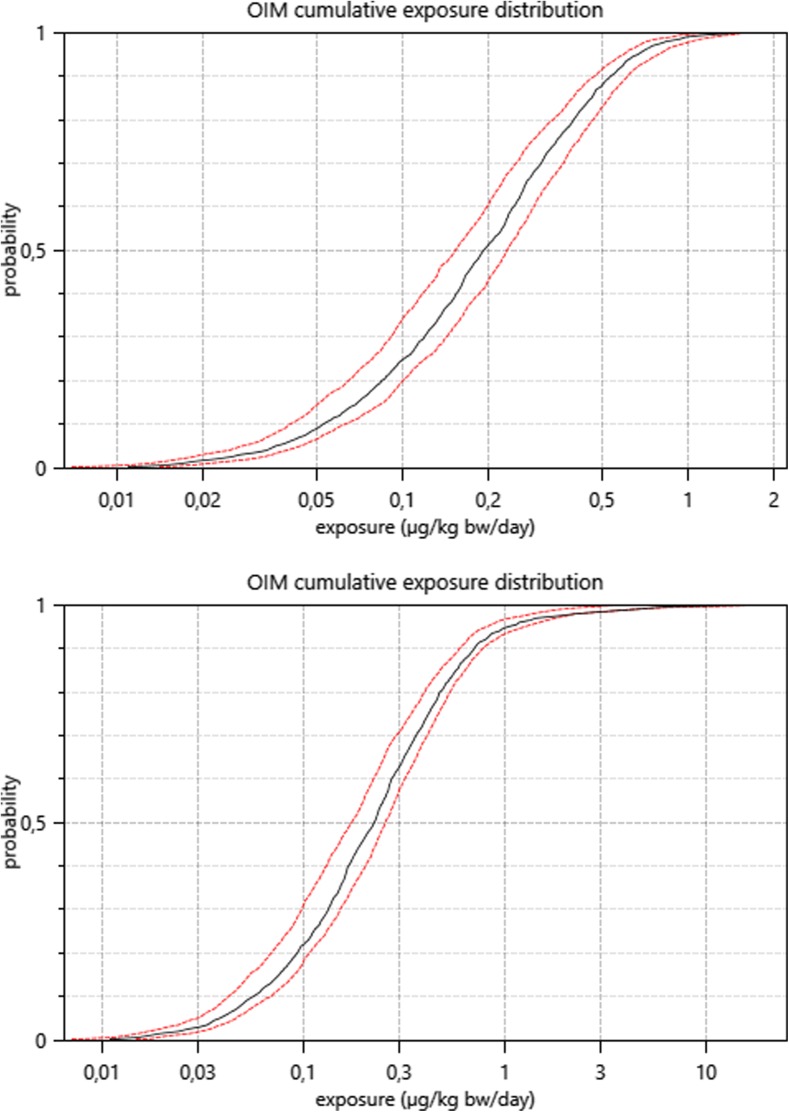
Cumulative distribution of dietary exposures (top panel) or aggregate exposures (bottom panel), with uncertainty intervals shown as dashed lines. OIM is the observed individual mean, selected in the MCRA run. This is an empirical calculation of expected daily exposures, per individual in the dietary survey

Table 10 shows estimated exposures for various population percentiles (similar to Table 4) together with 2.5–97.5% uncertainty bounds. Results based on the Bream2 non-dietary exposure model also include the uncertainty bounds. For comparison, results using the Browse model without uncertainty are also shown in Table 10. The mean dietary and aggregate exposure estimates are 0.26 (0.22–0.30) and 0.40 (0.31–0.44), estimated from 100 uncertainty loops. Uncertainty is greatest in the high percentile individuals. These are the individuals that are more likely to be affected by the non-dietary source. Due to random simulation in the uncertainty loops, the lower percentile estimates are also increased when non-dietary exposure is included. However, the effect from crop spraying is relatively small for the lower percentiles. A visual indication of the uncertainty is also seen from the graphs output from MCRA (Fig. 5). These again show how the upper tail of the distribution is affected more than the lower percentiles if the crop-spraying exposures are included in the assessment. Bream2 and Browse lead to broadly similar results. The greatest difference is seen in the 99.99th percentile.

**Table 10:** MCRA percentile calculations for dietary and aggregate chronic exposures within the UK adult population, based on the reference compound flusilazole. Uncertainty intervals (2.5–97.5%) are also shown. Aggregate exposures based on Bream2 and Browse are both included

Percentage	Dietary exposure (μg/kg bw/day)	Aggregate exposure (μg/kg bw/day) Bream2	Aggregate exposure (μg/kg bw/day) Browse
50	0.19 (0.15–0.23)	0.23 (0.17–0.26)	0.22
90	0.53 (0.47–0.61)	0.71 (0.60–0.78)	0.73
95	0.67 (0.59–0.79)	1.04 (0.82–1.25)	1.44
99	0.98 (0.87–1.22)	4.45 (1.87–4.31)	3.64
99.9	1.42 (1.17–1.56)	14.20 (3.53–16.25)	13.96
99.99	1.67 (1.27–2.09)	16.93 (5.43–23.07)	25.23

**Fig. 6:**
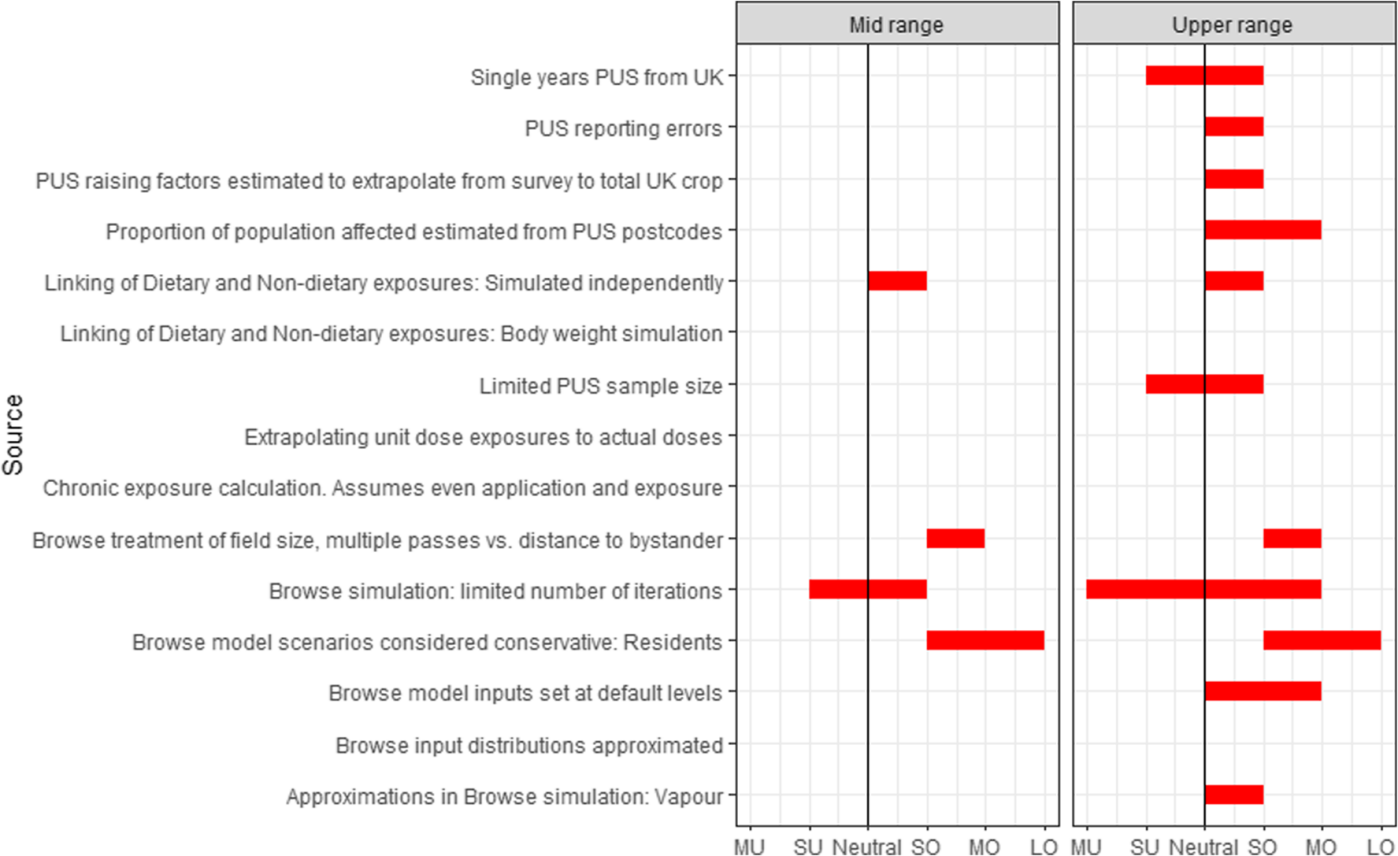
Summary of an informal subjective assessment of the impact of unquantified uncertainties, which are considered to have a potential effect on the estimates. Sources of unquantified uncertainties are listed on the vertical axis. Symbols on the horizontal axis represent (MU = medium underestimate; SU = small underestimate; SO = small overestimate; MO = medium overestimate; LO = large overestimate). Overestimation/underestimation relate to the mid-range (population median) or upper range (population 95th percentile) exposure estimates compared to the corresponding true exposure

Various simplifying assumptions and approximations have been made, and it is important to consider their potential impact on the outputs of the exposure model. Some of the main impacts are briefly discussed in Section “Discussion”. A more detailed assessment is provided in Supplementary Material using the framework of EFSA (2012b). Figure 6 shows a visualisation of this assessment using one of the communication tools presented in Tennant et al. (2017). Two features are worth noting: It reflects the belief that the unquantified uncertainties listed have more impact on the estimates of upper range exposures/individuals than on the exposure estimate of a typical individual. It also shows that for most of these factors, the estimates are considered to lead to overestimates of the true exposures. This is useful as a communication tool and to document the type of features that may be improved in future assessments.

## Discussion

Aggregate exposure assessments that allow for mixtures in multiple exposure pathways have been integrated within the MCRA software as part of recent EU projects Acropolis and Euromix. These tools can provide important information about realistic exposure patterns to risk assessors. The results presented above have demonstrated the use of the MCRA software to implement these methods using non-dietary exposure scenarios generated from two alternative exposure models for residents exposed to mixtures from crop spraying (Browse and Bream2). These case studies show the flexibility of MCRA to allow non-dietary exposure from external calculations of different types, for a particular exposure source. Alternative sources of non-dietary exposure, including bisphenols in cosmetic and other consumer products, are being considered separately using the same system (Karrer et al. submitted). Other sources of exposure could be included if appropriate models are available. The non-dietary model must generate an output file format that matches the MCRA non-dietary exposure survey format (referred to in section “Linking non-dietary exposures and dietary exposures in MCRA”). Substance types other than pesticides can be used within a cumulative and aggregate assessment implemented in MCRA provided the appropriate RPF values are included.

With Bream2, it was also illustrated how uncertainty and variability can be propagated through the aggregate model. The ability to separately present variability and uncertainty within the output is important in decision-making as it can influence the recommended course of action, specifically whether it is useful to obtain further information to reduce uncertainty or to introduce measures that might reduce variability of exposure. The Browse model does not separate uncertainty and variability, but rather combines uncertainty and variability about parameters into a single non-dietary exposure distribution. MCRA can aggregate using this simpler form, although the more detailed separation of uncertainty and variability is then lost in the resulting aggregate distribution.

The range of outputs presented in section “Dietary and aggregate exposure” illustrates how MCRA can give information about different aspects of the distribution of cumulative and aggregate exposures within a population. Further options to investigate sub-populations are available. This represents an extension of the tools available within the EU for dietary and non-dietary risk assessment.

Browse input parameters are by default set at conservative values. These include drift reduction (none) and absorption factors (100%). The calculation of chronic exposure takes an aggregated total spray over a year’s data per field and compound then uses a daily average over a 3-month period. This was intended to represent average daily exposure during a spraying season. A worst-case scenario might be an intensive spray period of only 7 days, but this would depend on the health endpoint being considered. A 3-month average is already more conservative than the daily average calculated over a full year. Dividing the total aggregate spray by 12 months rather than 3 would reduce the exposure by a factor of 4.

A more detailed assessment of risk should include a more accurate number of individuals living around each field and also a more complete list of UK grown crops. It should also consider the fact that field sizes and crop types will not be homogeneously dispersed and affecting equal proportions of the population. More complex correlation structures could be represented if it were known how many people live amongst fields of specific crop combinations, but such information is currently unavailable. The assumption of one crop per individual is considered a more realistic scenario than allowing individuals to live near a combination of crops, especially as Browse assumes the resident is always downwind and always present during spray applications. Note that the estimated number of individuals living near sprayed fields is approximately a factor of 5 higher than the previous study (CEH 2005) although both estimates are rather simplistic and use old data. In general, MCRA allows different absorption factors for the three routes (dermal, oral and inhalation) and for each compound if more precise information could be obtained about these.

Bodyweight simulations are generated as part of the Browse model, but these are not used in the aggregation of exposures. Instead, the bodyweight scaling used in MCRA is from the dietary consumption data and will therefore be independent of the non-dietary exposure.

Exposure for individuals living at 1000 m from the source has been estimated to be lower than those within 100 m of the sources (Wong et al. 2017) although there would be a larger group of people included. The Browse default direction of spray drift is directly towards the resident except for a small amount of random variation in the direction and includes three passes of the spray boom. In reality, there may be fewer passes, in which case the assessment overpredicts true exposure. The number of upwind passes is a relevant input that could be varied in a sensible way as a function of sprayed area, but this is not considered in the analysis presented here. Subsequent passes are 24 m further away from the resident and therefore contribute little to the total exposure.

The simplifications listed above lead to a more protective, conservative exposure assessment. As part of an aggregate exposure assessment that also includes dietary exposure sources, the levels of conservatism should be broadly similar, so that the overall level of conservatism is not dominated by a single source. The dietary exposure assessment also included conservative assumptions including the use of observed individual mean (OIM) dietary intake model. However, the residue model was based on the ‘EFSA optimistic scenario’. The EFSA pessimistic scenario leads to levels of exposure that are considered implausible. Currently, there is no practical method to ensure an equivalent level of conservatism.

The Browse runs used in the current analysis assume a single resident living long term adjacent to fields of a single crop type. This is a necessary simplification given the lack of realistic information about resident numbers in relation to different farm types. More complex scenarios could be designed if more information became available.

Unfortunately, it was not possible to compare the modelled aggregate results against real exposures such as biomonitoring data in the current study. This would require targeted sampling of UK residents living near sprayed fields and measurements of the 144 pesticides in their system. These data were not available.

As mentioned in section “Linking non-dietary exposures and dietary exposures in MCRA”, the estimation of RPFs to weight pesticides assumes dose-additivity as a simple but generally conservative model for cumulative risk assessment. This is the default currently implemented in MCRA. It would therefore not be possible to modify the results if there was evidence of synergistic or antagonistic effects within a given group of pesticides. However, if such evidence were obtained, its impact on the risk assessment should also be assessed as part of the uncertainty analysis. The use of RPFs will not be appropriate for all types of health effects and compound classes.

## Electronic supplementary material

ESM 1 (DOCX 34 kb)

## Footnotes

1 To simplify the description, amount of pesticide is used to refer to the amount of active ingredient in a pesticide product.
