# Supplementary material for: Modelling aggregate exposure to pesticides from dietary and crop spray sources in UK residents
Source: Environ Sci Pollut Res Int. 2019 Feb 8;26(10):9892–907. doi: 10.1007/s11356-019-04440-7 (PMC6469822; doi:10.1007/s11356-019-04440-7)
Supplement: Supplementary file 1 — (DOCX 34 kb) [file 11356_2019_4440_MOESM1_ESM.docx]

# Modelling aggregate exposure to pesticides from dietary and crop spray sources in UK residents – Supplementary materials for Environmental Science and Pollution Research

Marc C. Kennedy (Fera Science Limited, [Marc.kennedy@fera.co.uk](mailto:Marc.kennedy@fera.co.uk))

### S1 Unquantified uncertainties

#### S1.1 Resident modelling of non-dietary exposure using Browse

The following table lists those aspects of the true exposure for which uncertainties could not be quantified as part of the modelling process. It is still important to recognise these uncertainties and to give some indication of the level of overestimation or underestimation that will occur as a result. Here we consider only those aspects related to the non-dietary exposure or the aggregation process. A separate uncertainty table was developed for an example of dietary exposure (Boon et al., 2014). Some other uncertainties will be shared between dietary and non-dietary parts, such as definitions of CAG, toxicology, and absorption factors. The assessments here are approximate and subjective. They are listed here to illustrate the process recommended in EFSA (2012) and Tennant et al (2017). We decided not to add a numerical scale to the +, - symbols as this would give the impression of more accuracy than we can properly assess.

Table 1: An assessment of the impact of unquantified uncertainties, based on the approach suggested in EFSA (2012). Separate judgements are provided for the possible over- or under-estimation relative to the true population median exposure or the high level (P95) exposure. Symbols

| Source of uncertainty | Details | Assessment component | Impact on median exposure (P50 individual) | Impact on P95 individual exposure | Brief explanation |
| --- | --- | --- | --- | --- | --- |
| Data | Pesticide Usage | Limited PUS sample size | . | -/+ | Surveys are stratified, etc. to be representative of the main spraying activities and crop types in each UK region |
| Data | Pesticide Usage | Single year’s PUS from UK | . | -/+ | Does not account for temporal variation in actual spray patterns |
| Data | Pesticide Usage | PUS reporting errors | . | ./+ | May be measured incorrectly or included unused product amounts |
| Data | Pesticide Usage | PUS raising factors estimated to extrapolate from survey to total UK crop | . | ./+ |  |
| Data | Missing data required to extrapolate to population | Proportion of population affected estimated from PUS postcodes | . | ./++ | Little information available about the numbers of individuals living adjacent to sprayed fields of each crop type; impact of individuals living at distances beyond 20m are not modelled by Browse; Male/female residents treated the same |
| Model | Simplification in Browse | Chronic exposure calculation. Assumes even application and exposure | . | . | Assume that the total amount sprayed in a field impacts an individual evenly throughout the year. Timing of multiple applications is not considered. Limited impact on chronic exposure, due to averaging. |
| Model | Post-processing Browse outputs | Extrapolating unit dose exposures to actual doses | . | . | Simplified linear relationship assumed for computational reasons. Browse model is approximately linear in many cases, so limited impact |
| Model | Simplification in Browse | Approximations in Browse simulation: Vapour | . | ./+ | Single representative regions modelled, but at the conservative end of average temperature. This will only impact the (relatively small) values of inhalation |
| Model | Browse inputs | Browse model inputs set at default levels | . | ./++ | The default inputs were generally selected to be conservative (overestimation) for the general population but less so for a highly exposed individual. However, the median non-dietary exposure is unaffected due to the low percent of residents near farms |
| Model | Browse inputs | Browse input distributions approximated | . | . | To approximate population variation, some standard distributions have been fitted to data or parameter estimates |
| Model | Simplification in Browse/Browse inputs | Browse model scenarios considered conservative: Residents | +/+++ | +/+++ | Residents assumed to live between fields being sprayed with wind blowing directly towards them from both sides and with same product |
| Model | Simplification in Browse | Browse treatment of field size, multiple passes vs. distance to bystander | +/++ | +/++ | Simplified multiplicative model allows for multiple upwind passes but this is not included in the MCRA inputs. Very large square fields would not have the same exposure potential, relative to their size, as a small one, because individuals could only be close to one of the outer edges. |
| Simulation | Browse Monte Carlo settings | Browse simulation: limited number of iterations | -/+ | --/++ | Some numerical error, particularly in extreme tails |
| Simulation | Browse/MCRA link | Linking of Dietary and Non-dietary exposures: Body weight simulation | . | . | Unrealistic assumption that body weight independent of breathing rates. Small impact as inhalation is estimated to be much smaller than dermal exposure |
| Simulation | Browse/MCRA link | Linking of Dietary and Non-dietary exposures: Simulated independently | ./+ | ./+ | No account taken of realistic consumption patterns, time spent at home, or differences between the dietary survey population and the residents living near farms |
|  |  | **Combined effects** | **./+** | **+/++** | **Overall, we consider that exposure will be over-estimated by factor around 5 – 10 in the upper tail but much less in the median estimate as most individuals do not live next to a farm** |

Key +++ large overestimation; ++ medium overestimation; + small overestimation; . Neutral, i.e. no substantial over or underestimation; - small underestimation; -- medium underestimation; --- large underestimation. Symbols separated by / represent ranges, e.g. +/- means that the estimate is believed to be between a small underestimation or small overestimation.

## References

Boon PE, van Donkersgoed G, Christodoulou D, Crépet A, D'Addezio L, Desvignes V, Ericsson B-G, Galimberti F, Ioannou-Kakouri E, Jensen BH, Rehurkova I, Rety J, Ruprich J, Sand S, Stephenson C, Stromberg A, Turrini A, van der Voet H, Ziegler P, Hamey P, van Klaveren JD (2014) Cumulative dietary exposure to a selected group of pesticides of the triazole group in different European countries according to the EFSA guidance on probabilistic modelling. Food Chem. Toxicol. 79:13-31

EFSA (2012). Guidance on the use of probabilistic methodology for modelling dietary exposure to pesticide residues. EFSA Journal 2839:95.

Tennant D, Bánáti D, Kennedy MC, König J, O’Mahony C, Kettler S (2017) Assessing and Reporting Uncertainties in Dietary Exposure Analysis – Part II: Application of the uncertainty template to a practical example of exposure assessment. Food and Chemical Toxicology, 109:68-80
